# Overexpression of circRNA SNRK targets miR-103-3p to reduce apoptosis and promote cardiac repair through GSK3β/β-catenin pathway in rats with myocardial infarction

**DOI:** 10.1038/s41420-021-00467-3

**Published:** 2021-04-19

**Authors:** Yeqian Zhu, Pengcheng Zhao, Ling Sun, Yao Lu, Wenwu Zhu, Jian Zhang, Chengyu Xiang, Yangming Mao, Qiushi Chen, Fengxiang Zhang

**Affiliations:** 1 Section of Pacing and Electrophysiology, Division of Cardiology, the First Affiliated Hospital with Nanjing Medical University, Nanjing, China; 2 Department of Cardiology, the Affiliated Changzhou No. 2 People’s Hospital of Nanjing Medical University, Changzhou, China

**Keywords:** Myocardial infarction, Apoptosis

## Abstract

Ischemic cardiomyopathy seriously endangers human health leading to a poor prognosis. Acute myocardial infarction (AMI) is the primary etiology, and the pathophysiological process concludes with the death of cardiomyocytes caused by acute and persistent ischemia and hypoxia in the coronary arteries. We identified a circRNA (circSNRK) which was downregulated in rats with myocardial infarction (MI), however, the role it plays in the MI environment is still unclear. This study contained experiments to investigate the role of circSNRK in the regulation of cardiac survival and explore the mechanisms underlying circSNRK functions. Quantitative real-time PCR (qRT-PCR) was performed to determine the circSNRK expression patterns in hearts. Gain-of-function assays were also conducted in vitro and in vivo to determine the role of circSNRK in cardiac repair. qRT-PCR, western blot, and luciferase reporter assays were used to study circRNA interactions with micro RNAs (miRNAs). Overexpression of circSNRK in cardiomyocytes reduced apoptosis and increased proliferation. Adeno associated virus 9 (AAV9) mediated myocardium overexpression of circSNRK in post MI hearts reduced cardiomyocyte apoptosis, promoted cardiomyocyte proliferation, enhanced angiogenesis, and improved cardiac functions. Overall, upregulation of circSNRK promotes cardiac survival and functional recovery after MI. Mechanistically, circSNRK regulates cardiomyocyte apoptosis and proliferation by acting as a miR-103-3p sponge and inducing increased expression of SNRK which can bind GSK3β to regulate its phosphorylated activity. And thus circSNRK may be a promising therapeutic target for improving clinical prognosis after MI.

## Introduction

Ischemic cardiomyopathy seriously endangers human health leading to a poor prognosis^1,2^. Acute myocardial infarction (AMI) is the primary etiology, and the pathophysiological process concludes with the death of cardiomyocytes caused by acute and persistent ischemia and hypoxia in the coronary arteries^3,4^. Previous studies have revealed that a reduction in cardiomyocyte apoptosis and activation of cardiomyocyte proliferation present promising opportunities for limiting the loss cardiomyocytes after MI^5,6^. However, these strategies offer cardiomyocytes limited protection from MI injury, thus finding novel molecular entities and developing innovative effective therapies for MI are of critical importance.

CircRNAs (circular RNAs) are a novel class of non-coding RNAs that form a covalently closed continuous loop. They are a current focus of research in the field of RNA, and support many gene regulatory functions as discovered in recent years^7^. Compared with linear mRNA, circRNAs are circularized by a downstream 5′ splice site to an upstream 3′ splice site^8,9^. CircRNAs expression is more stable and resistant to degradation by exonucleases and their circular structure maintains their gene regulatory roles in binding to and inhibiting microRNAs (miRNAs)^8,10,11^. Certain kinds of circRNAs have been found participate in cardiac development and physiology^12,13^. A recent study suggested that knockdown of circNCX1 could reduce the levels of CDIP1 and attenuate apoptosis and I/R injury^14^. Another study demonstrated that loss of circNfix improves cardiac regenerative repair post MI by suppressing Ybx1 ubiquitin-dependent degradation and increasing miR-214^15^. Therefore, targeting circRNAs has the potential to be an effective strategy to protect cardiac function.

**Fig. 1:**
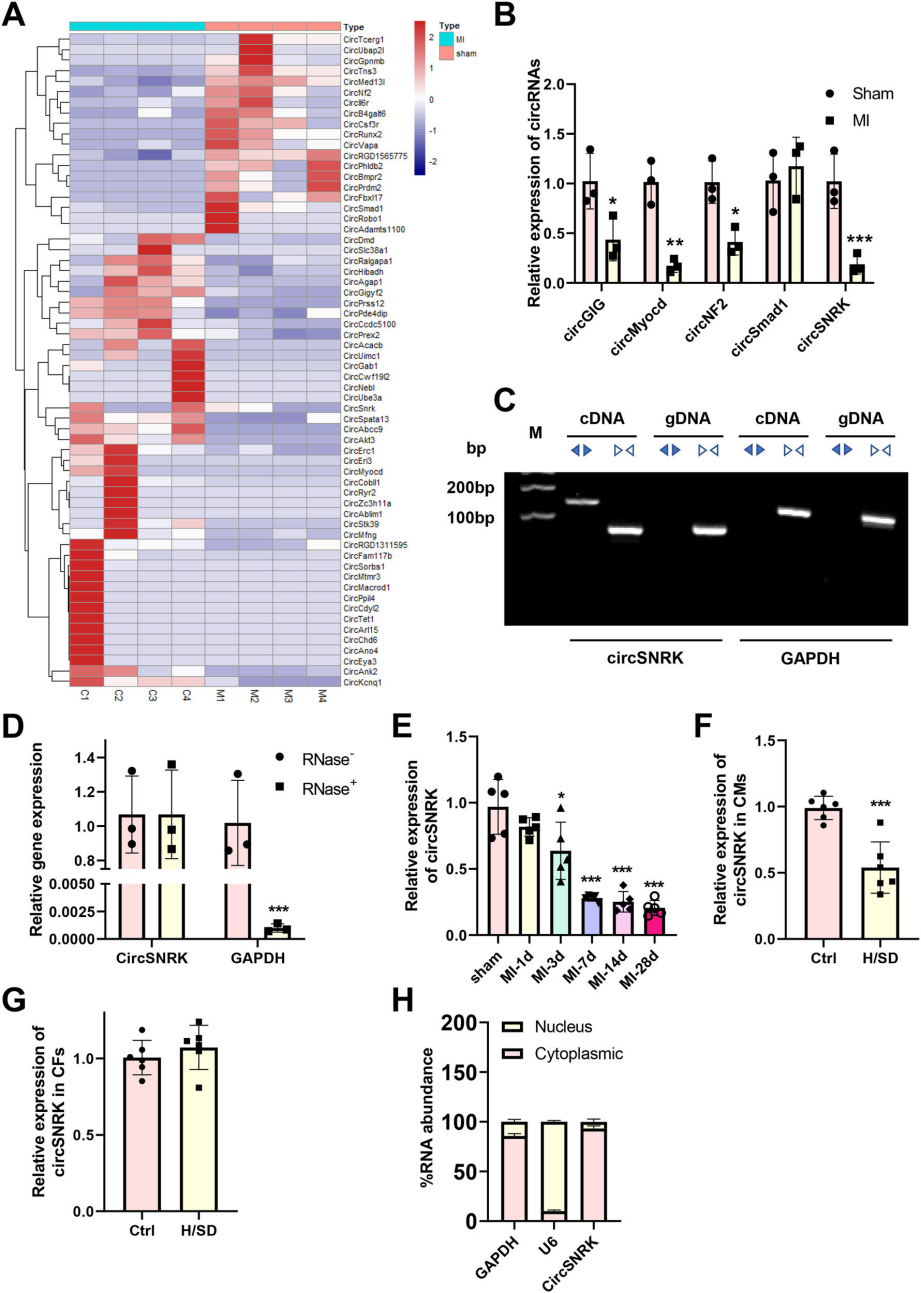
**CircRNA profiling in MI hearts**. **A** Heat map of circRNAs differentially expressed at 3 days post MI. [C1, C2, C3, and C4] denotes expression in sham animals while [M1, M2, M3, and M4] denotes animals subject to MI. Relative expression values are indicated on the color key and increase in value from navy to firebrick (*n* = 4). **B** Quantitative real-time PCR (qRT-PCR) analysis of differentially expressed circRNAs (*n* = 3). **C** Divergent primers amplify circSNRK in cDNA but not genomic DNA (gDNA); divergent and convergent primers are indicated by the direction of the arrow. **D** The abundances of circSNRK and GAPDH mRNA in cardiomyocytes treated with RNase R, which were normalized to the amount measured in the mock treatment. (*n* = 3). **E** qRT-PCR analysis of circSNRK expression at different time points in post MI heart tissues (*n* = 5) compared to sham (*n* = 5). **F**, **G** qRT-PCR analysis of circSNRK in isolated cardiomyocytes and fibroblasts from neonatal rat hearts under normal and H/SD conditions. (*n* = 6). **H** qRT-PCR for the abundance of circSNRK in either the cytoplasm or nucleus of primary CMs. *<0.05, **P < 0.01, ***p < 0.001.

However, it remains unknown whether regulation of circRNAs can promote cardiac function post MI. In the current study, total RNA was isolated from sham or MI rat hearts 3 days after MI to perform high throughput sequencing analysis. The results showed that sixty-six circRNAs were differentially expressed with 19 upregulated and 47 downregulated in the post-MI hearts compared to sham hearts (Fig. 1A). Of them, overexpression of circSNRK was found to reduce cardiomyocyte apoptosis, promote cardiomyocyte proliferation, enhance angiogenesis, and promote cardiac function post-MI in rat.

## Results

### CircRNA profiling in MI hearts

High throughput sequencing was used to validate that several circRNAs were expressed, including the circSNRK that was significantly downregulated in the MI hearts compared to sham groups (Fig. 1B). Using divergent primers, circSNRK was amplified in cDNA but not in genomic DNA, indicating that this RNA species is circular in form (Fig. 1C). RNase R treatment was then used to induce the degradation of GAPDH mRNA, but had no effect on the closed covalent structure of circSNRK, confirming that circSNRK had greater stability than the linear mRNA (Fig. 1D). These results suggest that circSNRK plays a key role in the pathophysiology of MI. Therefore, we focused on circSNRK for expression pattern and functional characterization. Upon examining circSNRK expression in rat hearts at different time after MI, we demonstrated that it continued to decrease throughout the follow-up of 7 days post MI compared to sham hearts (Fig. 1E). Next, we examined where circSNRK is expressed in the post-MI hearts: circSNRK was dramatically downregulated in cardiomyocytes, but not in fibroblasts (Fig. 1F, G). With >85% homology among humans and rats, circSNRK nucleotide sequence is strongly conserved, (Supplementary Fig. 1A). In addition, according to the result of nucleo-cytoplasmic separation assay, circSNRK locates mainly in the cytoplasm of cardiomyocytes (Fig. 1H).

### CircSNRK elevation ameliorates cardiomyocyte apoptosis and promotes cardiomyocyte proliferation in vitro

**Fig. 2:**
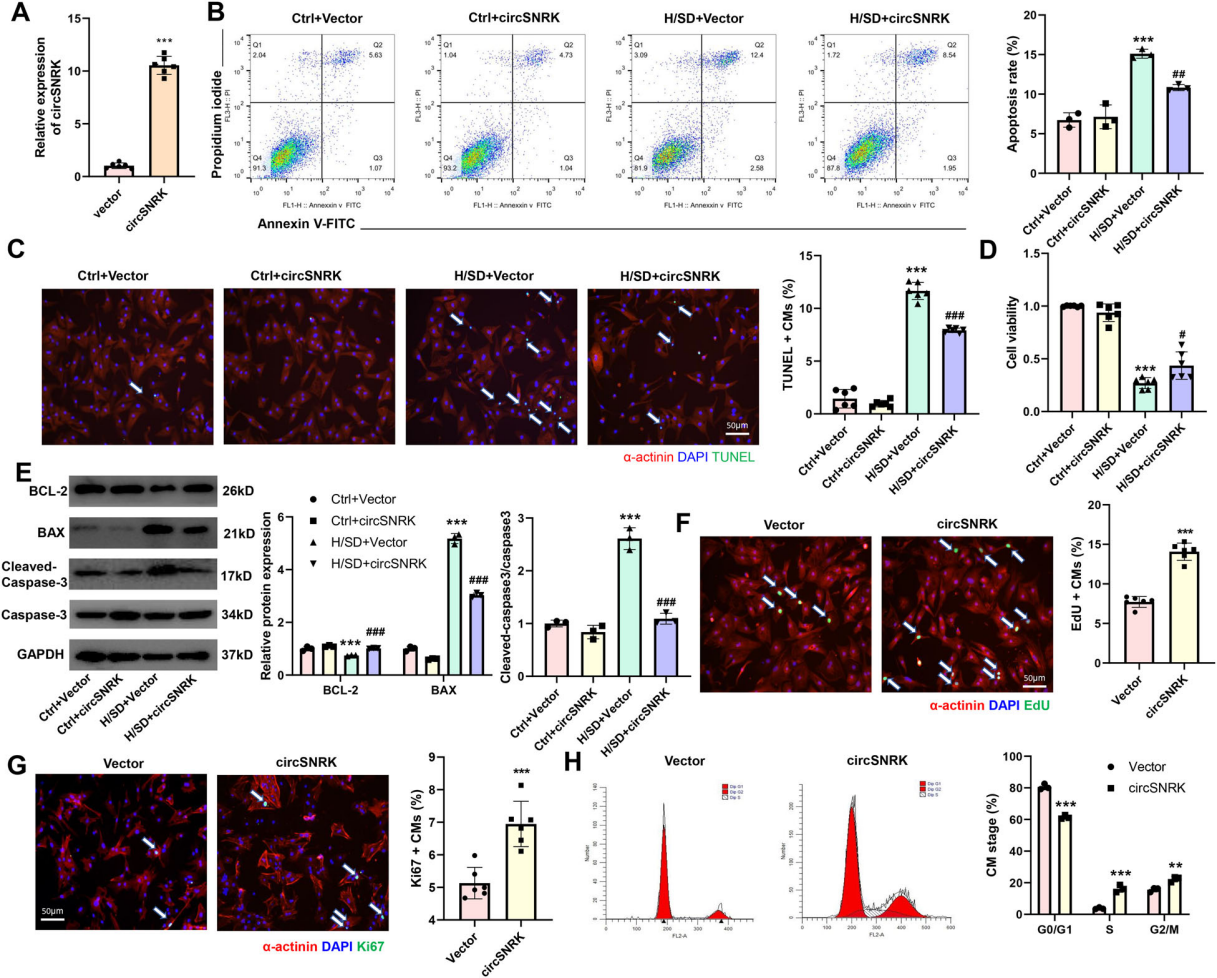
**CircSNRK elevation ameliorates cardiomyocyte apoptosis and promotes cardiomyocyte proliferation in vitro**. **A** qRT-PCR analysis of circSNRK in vector or circSNRK overexpression plasmid treated primary cardiomyocytes. ***p < 0.001 vs. vector group (*n* = 6). **B**–**D** Primary cardiomyocytes were transfected with vector and circSNRK overexpression plasmid in normoxia and H/SD conditions. CircSNRK reduced apoptosis by flow cytometry analysis (*n* = 3) (**B**) and TUNEL analysis. White arrows indicate apoptotic cells. Bars = 50 μm. (*n* = 6). **C** CircSNRK increased cell viability in H/SD condition by Cell Counting Kit-8 (CCK-8) assay. (*n* = 6). **D** ***p < 0.001 *vs*. Ctrl + Vector, ^#^*P* < 0.05, ^##^*P* < 0.01, ^###^*p* < 0.001 vs. H/SD + Vector. **E** BCL-2, BAX and cleaved-caspase-3/caspase-3 levels after overexpression of circSNRK in primary cardiomyocytes. ***p < 0.001 vs. Ctrl + Vector, ^###^*p* < 0.001 vs. H/SD + Vector (*n* = 3). **F** EdU staining in isolated cardiomyocytes transfected with Vector and circSNRK and quantification of EdU positive cardiomyocytes. White arrows indicate EdU positive CMs. ***p < 0.001 vs. Vector; bar = 50 µm. (*n* = 6). **G** Ki67 immunofluorescence staining in isolated cardiomyocytes transfected with Vector and circSNRK and quantification of Ki67 positive CMs. White arrows indicate Ki67-positive CMs. ***p < 0.001 vs. Vector; bar = 50 µm. (*n* = 6). **H** Flow cytometry analysis of primary cardiomyocytes transfected with Vector and circSNRK. **P < 0.01, ***p < 0.001 versus Vector group (*n* = 3).

To investigate the function of circSNRK in cardiovascular biology, we used circSNRK overexpression plasmids that were transiently transfected into neonatal rat cardiomyocytes. Delivery of circSNRK overexpression plasmid significantly increased primary cardiomyocyte circSNRK levels (Fig. 2A). After the plasmids were transfected for 24 h, the primary cardiomyocytes were exposed to hypoxia and ischemia for another 12 h. Flow cytometry showed that circSNRK decreased the apoptosis of primary cardiomyocytes in H/SD conditions (Fig. 2B). In addition, the numbers of TUNEL positive cells decreased in primary cardiomyocytes expressing circSNRK (Fig. 2C). We also demonstrated that circSNRK overexpression restrained H/SD induced reduced cell viability (Fig. 2D). Under H/SD conditions, the ratio of cleaved-caspase-3/caspase3 and BAX were depressed but BCL-2 expression was enhanced by circSNRK up-regulation (Fig. 2E). These in vitro findings suggest that increased the expression of circSNRK confers the resistance of cardiomyocytes to hypoxic and ischemic insult. Next, the effects of circSNRK overexpression on cardiomyocytes proliferation were explored. Upregulation of circSNRK was found to increase the ratio of EdU and Ki67 positive primary cardiomyocytes (Fig. 2F, G). There were more primary cardiomyocytes accumulating at the synthesis and gap2 phases of the cell cycle by flow cytometry assays (Fig. 2H). These results suggested that circSNRK expression might be associated with cardiomyocyte proliferation.

### CircSNRK exerts its cardioprotective effect by sponging miR-103-3p

**Fig. 3:**
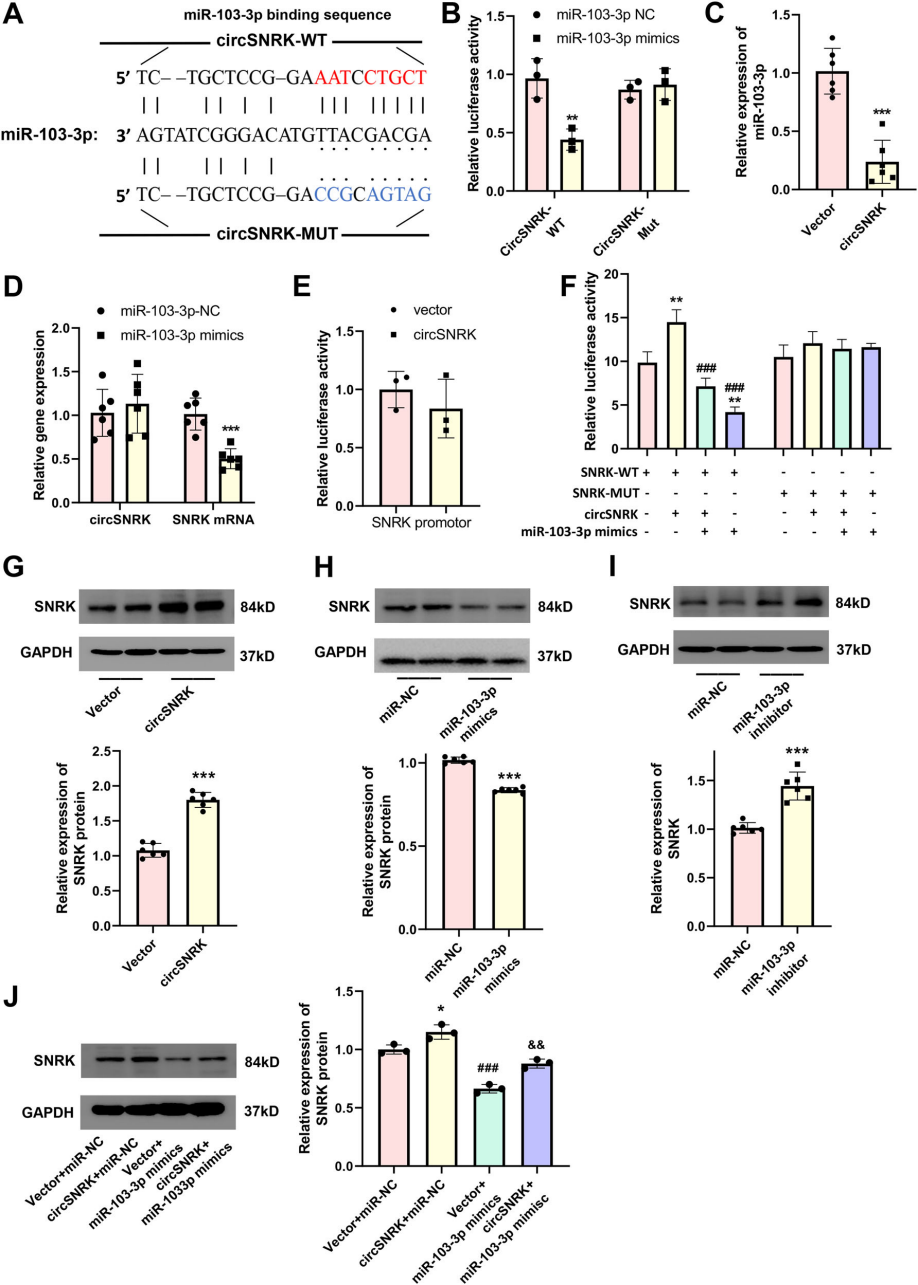
**CircSNRK exerts its cardioprotective effect by sponging miR-103-3p**. **A** Graphical representation showing the predicted sites of miR-103-3p for binding to circSNRK and the corresponding mutation. **B** Luciferase activity of primary cardiomyocytes transfected with luciferase-circSNRK-WT and luciferase-circSNRK-Mut. **P < 0.01 vs. miR-103-3p*-*NC. (*n* = 3). **C** qRT-PCR analysis of miR-103-3p in vector or circSNRK overexpression plasmid treated primary cardiomyocytes. ***p < 0.001 vs. vector (*n* = 6). **D** qRT-PCR analysis of circSNRK and SNRK mRNA in miR-NC or miR-103-3p treated primary cardiomyocytes. ***p < 0.001 vs. vector (*n* = 6). **E** Luciferase reporter assay was used to test the luciferase activity of SNRK promoter region (*n* = 3). **F** Luciferase activity of primary cardiomyocytes transfected with luciferase-SNRK 3′ UTR-WT and luciferase-SNRK 3′ UTR-Mut. **P < 0.01 vs. miR-103-3p-NC + SNRK-WT, ^###^*P* < 0.001 versus miR-103-3P-NC + circSNRK+ SNRK-WT (*n* = 3). **G** SNRK protein level after overexpression of circSNRK. ***p < 0.001 vs. vector (*n* = 6). **H** SNRK protein level after overexpression of miR-103-3p. ***p < 0.001 vs. miR103-3p-NC (*n* = 6). **I** SNRK protein level after downregulation of miR*-*103-3p. ***p < 0.001 vs. miR-103-3p-NC (*n* = 6). **J** SNRK protein level after circSNRK and miR-103-3p interference in cardiomyocytes. *P < 0.05 vs. Vector + miR-NC, ^###^*P* < 0.001 vs. circSNRK + miR-NC, ^&&^*P* < 0.01 vs. circSNRK + miR-103-3p mimics (*n* = 3).

Two miRNAs that may interact with circSNRK were found using bioinformatics prediction analysis and the RNA22 (https://cm.jefferson.edu/data-tools-downloads/rna22-full-sets-of-predictions/). MiR-103-3p and miR-103-1-5p had the most binding sites on circSNRK, however, miR-103-1-5p is poorly conserved. In addition, miR-103-3p has also been implicated in cell apoptosis^16^ and proliferation^17,18^. We explored whether the cardioprotective effects of circSNRK was involved in its sponging to miR-103-3p and miR-103-3p’s target genes. We demonstrated that miR-103-3p was upregulated in MI myocardium (Supplementary Fig. 1B). The results of the luciferase assay confirmed the binding of miR-103-3p on circSNRK (Fig. 3A, B). CircSNRK overexpression significantly decreased miR-103-3p expression levels (Fig. 3C). QRT-PCR result showed that the miR-103-3p mimics obviously decreased SNRK expression but did not change the expression of circSNRK in CMs (Fig. 3D). Subsequently, we also found that the luciferase activity of SNRK promoter region presented no significant difference after overexpressing circSNRK (Fig. 3E). It further confirmed that circSNRK didn’t engage in regulating transcription. Moreover, the binding site of miR-103-3p in the 3′UTR of SNRK is highly conserved across species, suggesting SNRK as a common target gene of miR-103-3p. SNRK 3′UTR was cloned into a luciferase vector and then transfected into 293T cells. It was found reduced luciferase activity via transfection with miR-103-3p mimics. Furthermore, circSNRK increased luciferase activity, which could be abrogated by miR-103-3p mimics (Fig. 3F). Similarly, SNRK protein expression was significantly increased transfected with circSNRK compared with that in cardiomyocytes transfected with circSNRK plasmid (Fig. 3G). SNRK protein expression was dramatically downregulated by miR-103-3p mimics but upregulated by miR-103-3p inhibitor (Fig. 3H, I), and miR-103-3p mimics abolished the effect of circSNRK overregulation on SNRK (Fig. 3J). These results revealed that circSNRK only acted as a sponge for miR-103-3p and impaired miR-103-3p activity via sequestering miR-103-3p from its target genes.

### miR-103-3p elevation aggravates H/SD induced cardiomyocyte apoptosis and reduced cardiomyocyte proliferation

The role of miR-103-3p in H/SD-induced cardiomyocytes apoptosis was explored. We overexpressed miR-103-3p in primary cardiomyocytes with miR-103-3p mimics (Supplementary Fig. 2A). MiR-103-3p overexpression increased the apoptosis of cardiomyocytes in vitro (Supplementary Figs. 2B–4D). Then, apoptosis-related proteins were detected, the results showed that protein expression of BAX and the ration of cleaved-caspase-3/caspase3 significantly increased while the protein level of BCL-2 decreased in the miR-103-3p mimics group (Supplementary Fig. 2E). Then, the role of miR-103-3p on cardiomyocytes proliferation was investigated. We found that overexpression of miR-103-3p decreases the ratio of primary cardiomyocytes expressing proliferation markers (EdU and Ki67) (Supplementary Fig. 2F, G) and primary cardiomyocytes at the synthesis and g2 gap phases of the cell cycle (Supplementary Fig. 2H). Therefore, these results revealed that miR-103-3p generally augments apoptosis and reduces proliferation in cardiomyocytes in vitro.

### Loss of miR-103-3p ameliorates hypoxia-induced cardiomyocyte apoptosis and increased cardiomyocyte proliferation

Then, we explored whether loss of miR-103-3p could alleviate H/SD-induced cardiomyocytes injury. Knockdown of miR-103-3p by miR-103-3p inhibitors significantly decreased apoptosis rate and the numbers of TUNEL positive cells, and increased cell viability in primary cardiomyocytes (Supplementary Fig. 3A–C). Similar reductions in BAX expression and ratio of cleaved-caspase-3/caspase3 were observed by knockdown of miR-103-3p using miR-103-3p inhibitor (Supplementary Fig. 3D). In addition, the cell cycle activity of primary cardiomyocytes was found to increase by down-regulation of miR-103-3p (Supplementary Fig. 3E–G). In all, these findings showed that inhibition of miR-103-3p can reduce H/SD induced cardiomyocyte apoptosis and increase cardiomyocyte proliferation.

### The miR-103-3p-SNRK regulatory axis underlies cardioprotection by circSNRK

**Fig. 4:**
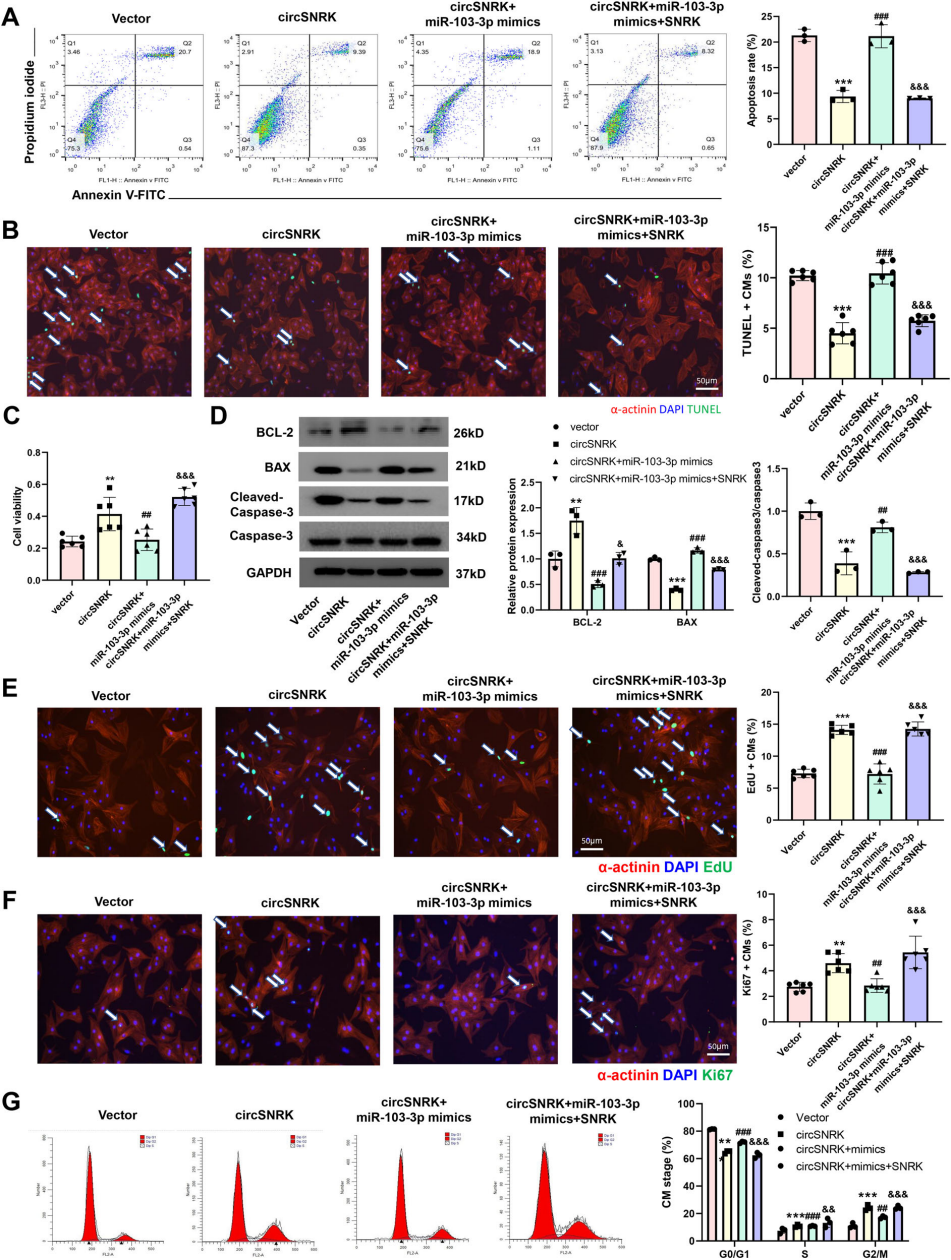
**The miR-103a-3p-SNRK regulatory cascade underlies cardioprotection by circSNRK**. **A**–**C** Primary cardiomyocytes were transfected with Vector, circSNRK, circSNRK + miR-103-3p mimics and circSNRK + miT-103-3p mimics + SNRK in H/SD conditions. Flow cytometry analysis (*n* = 3) (**A**) and TUNEL analysis (**B**) in primary cardiomyocytes after circSNRK, miR-103-3p, and SNRK interference. White arrows indicate apoptotic cells. bar = 50 μm. And Cell Counting Kit-8 (CCK-8) assay (*n* = 6). **C** **P < 0.01, ***p < 0.001 vs. Vector, ^##^*P* < 0.01, ^###^*p* < 0.001 vs. circSNRK, ^&&&^*p* < 0.001 vs. circSNRK + miR-103-3p mimics (*n* = 6). **D** BCL-2, BAX, cleaved-caspase-3/caspase-3 levels in hypoxic and ischemic primary cardiomyocytes after circSNRK, miR-103-3p, and SNRK interference. **P < 0.01, ***p < 0.001 vs. Vector, ^##^*P* < 0.01, ^###^*p* < 0.001 vs. circSNRK, ^&^*P* < 0.05, ^&&&^*p* < 0.001 vs. circSNRK + mimics (*n* = 3). **E** EdU staining in isolated NRCMs after circSNRK^,^ miR-103-3p, and SNRK interference and quantification of EdU positive primary cardiomyocytes. White arrows indicate EdU positive primary cardiomyocytes. ***p < 0.001 vs. Vector, ^###^*p* < 0.001 vs. circSNRK, ^&&&^*p* < 0.001 vs. circSNRK + miR-103-3p mimics; bar = 50 µm. (*n* = 6). **G** Ki67 immunofluorescence staining in isolated primary cardiomyocytes after circSNRK, miR-103-3p, and SNRK interference and quantification of Ki67 positive primary cardiomyocytes. White arrows indicate Ki67-positive primary cardiomyocytes. **P < 0.01 vs. Vector, ^##^*P* < 0.01 vs. circSNRK, ^&&&^*p* < 0.001 vs. circSNRK + miR-103-3p mimics; bar = 50 µm. (*n* = 6). **H** Flow cytometry analysis of primary cardiomyocytes transfected with miR-NC and miR-103-3p inhibitor. **P < 0.01, ***p < 0.001 vs. vector group, ^##^*p* < 0.01 ^###^*p* < 0.001 vs. circSNRK, ^&&^*p* < 0.01 ^&&&^*p* < 0.001 vs. circSNRK + miR-103-3p mimics (*n* = 3).

The above results suggested that the circSNRK-miR-103-3p-SNRK regulatory cascade is involved in cardiomyocytes. Then, we investigated whether such a regulatory axis was involved in regulating cardiomyocytes apoptosis and proliferation. Flow cytometry apoptosis assays, TUNEL staining assays, CCK-8 assay, and WB results revealed that miR-103-3p mimics impaired the effect of circSNRK overexpression on cardiomyocyte, which was restored by SNRK overexpression (Fig. 4A–D). This regulatory axis is also adaptive in cardiomyocytes proliferation, miR-103-3p mimics impaired the effect of circSNRK overexpression on proliferation, which was reversed by SNRK up-regulation (Fig. 4E–G). Overall, circSNRK plays a role as an endogenous miR-103-3p sponge to up-regulate SNRK expression in cardiomyocytes. The circSNRK-miR-103-3p-SNRK regulatory axis contributes to the cardioprotection.

### Overexpression of circSNRK ameliorates cardiac dysfunction after myocardial infarction

**Fig. 5:**
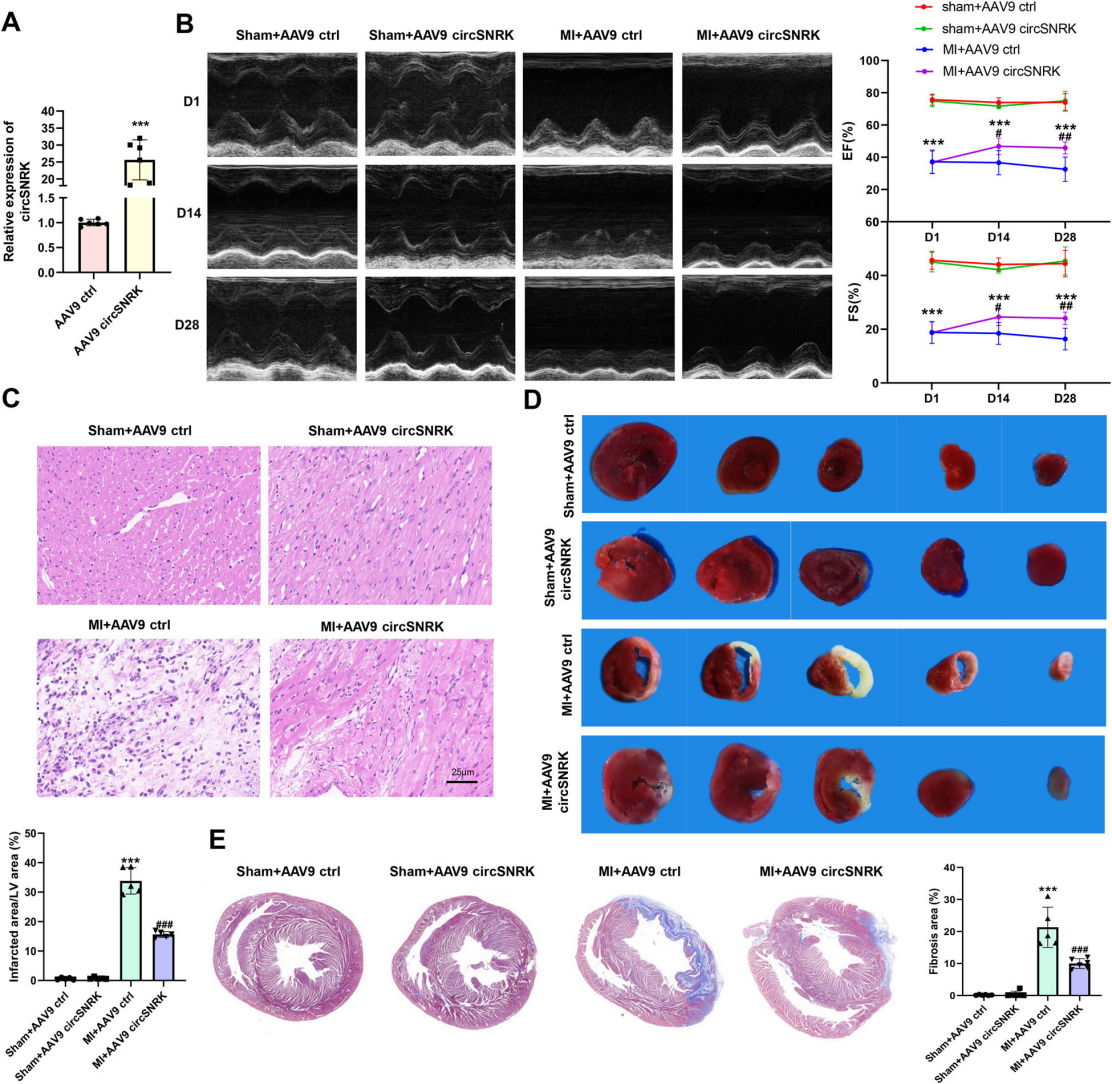
**Overexpression of circSNRK ameliorates cardiac dysfunction after myocardial infarction**. **A** QRT-PCR analysis of circSNRK in AAV9 ctrl and AAV9 circSNRK treated rat hearts. ***p < 0.001 vs. AAV9 ctrl group. **B** Echocardiography analysis was performed at 1day,14days and 28 days after establishment of MI. And quantitative analysis of LVEF and LVFS in the AAV9 circSNRK and AAV9 ctrl groups. ***p < 0.001 vs. Sham + AAV9 ctrl group; ^#^*p* < 0.05, ^##^*p* < 0.01 vs. MI + AAV9 ctrl group. (*n* = 5–6 for each group). **C** HE staining images at the border zone 28 days after MI. bar = 25 µm. **D** Triphenyltetrazolium chloride (TTC) staining of rat ventricular cross-sections at 28 days post-MI, ***P < 0.001 vs. Sham + AAV9 ctrl, ^###^*P* < 0.001 vs. MI + AAV9 ctrl. (*n* = 5 for each group). **E** Masson staining images at 28 days after MI and quantitative analyses of infarct size. Red, myocardium; blue, scarred fibrosis. ***p < 0.001 vs. MI + AAV9 ctrl group. ^###^*P* < 0.001 vs. MI + AAV9 ctrl. (*n* = 5–6 for each group).

Whether circSNRK has cardioprotective function in vivo, circSNRK gain-of-function studies were performed. We identified an obvious overexpression of circSNRK in ventricular myocardium at the fourth week post MI following intramyocardial injection of AAV9-circSNRK in the MI model (Fig. 5A). The alleviation of cardiac dysfunction by MI in rats expressing AAV9- circSNRK was found by echocardiographic analysis, as indicated by the higher LVFS and LVEF (Fig. 5B). Inflammatory cell infiltration also significantly decreased in the MI + AAV9 circSNRK group compared with the MI + AAV9 ctrl groups (Fig. 5C). Through TTC (triphenyltetrazolium chloride) staining and Masson trichrome staining, it showed that the infarcted area and cardiac fibrosis were dramatically reduced in AAV9 circSNRK injected rats compared with AAV9 ctrl injected rats (Fig. 5D, E). These results suggest that circSNRK is a crucial component of the cardioprotection in vivo.

### Overexpression of circSNRK reduces cardiomyocyte apoptosis and promotes adult cardiac regeneration after MI

**Fig. 6:**
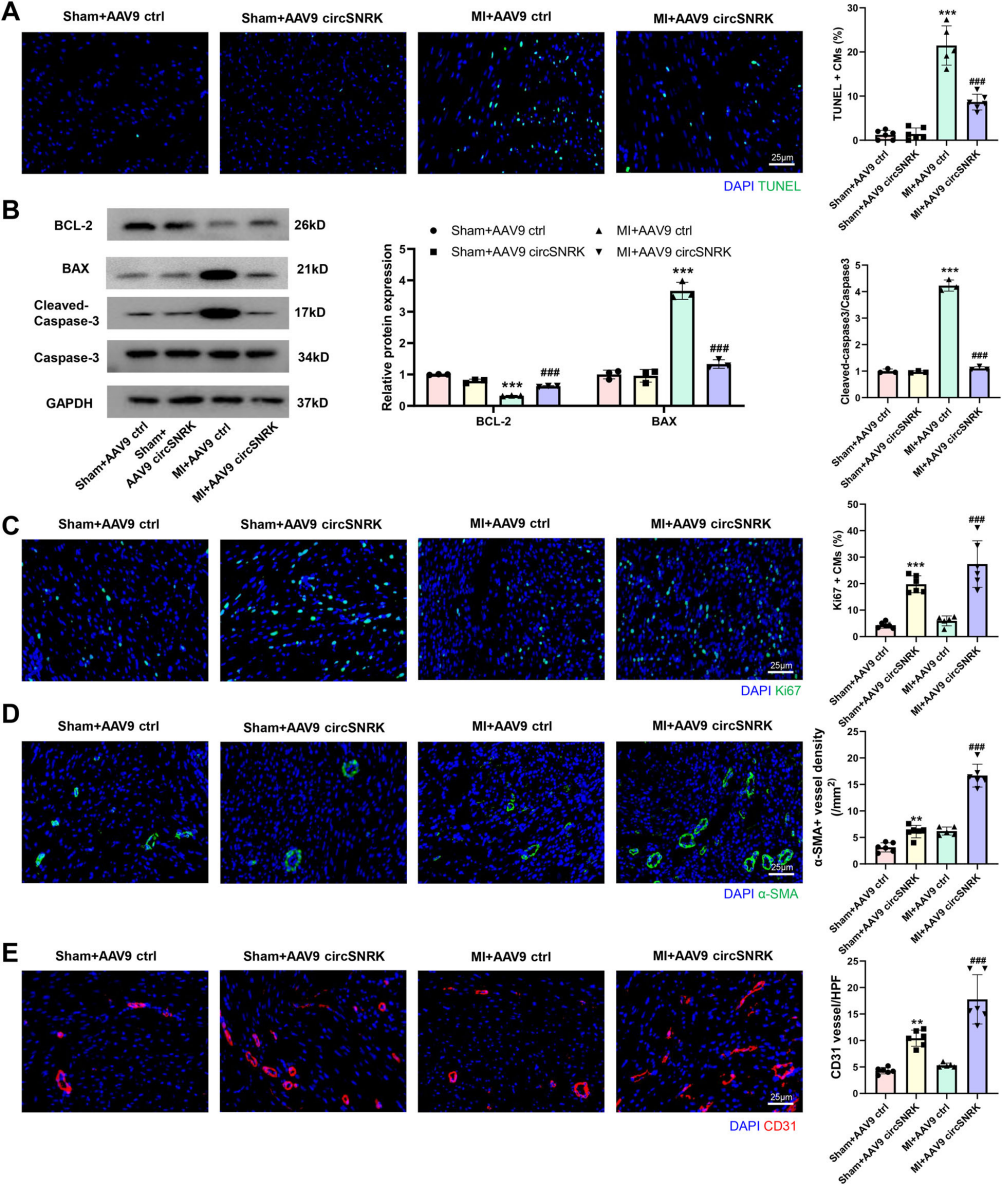
**Overexpression of circSNRK reduces cardiomyocyte apoptosis and promotes adult cardiac regeneration after MI**. **A** TUNEL immunofluorescence staining in the AAV9 ctrl and AAV9 circSNRK groups 7 days post MI and quantification of TUNEL positive CMs. ***p < 0.001 vs. Sham + AAV9 ctrl, ^###^*p* < 0.001 vs. MI + AAV9 ctrl; bar = 25 µm. (*n* = 5–6 for each group). **B** BCL-2, BAX, cleaved-caspase-3/caspase-3 levels in heart tissue after AAV9 ctrl and AAV9 circSNRK treatment. ***p < 0.001 vs. Sham + AAV9 ctrl, ^###^*p* < 0.001 vs. MI + AAV9 ctrl (*n* = 3). **C** Ki67 immunofluorescence staining in adult rat hearts 14 days after transfection with AAV9 circSNRK and AAV9 ctrl and quantification of Ki67-positive cardiomyocytes. ***p < 0.001 vs. Sham + AAV9 ctrl group; ^###^*p* < 0.001 vs. MI + AAV9 ctrl group. bar = 25 µm (*n* = 5–6 for each group). **D** α-SMA (α-smooth muscle actin) staining in the AAV9 ctrl and AAV9 circSNRK groups 14 days after transfection with AAV9 circSNRK and AAV9 ctrl and quantification of α-SMA-positive capillaries, ***p < 0.001 *vs*. Sham + AAV9 ctrl group; ^###^*p* < 0.001 vs. MI + AAV9 ctrl group. bar = 25 µm (*n* = 5–6 for each group). **E** CD31 staining in the border zone of ischemic hearts and quantification of CD31 positive blood vessels. **p < 0.01 vs. Sham + AAV9 ctrl group; ^###^*p* < 0.001 vs. MI + AAV9 ctrl group. bar = 25 µm (*n* = 5–6 for each group).

Apoptotic cells dramatically decreased at the border zone in AAV9 circSNRK rats compared with AAV9 ctrl rats (Fig. 6A). We then detected apoptosis-related proteins and found that protein expression of BAX and the ratio of cleaved-caspase-3/caspase-3 significantly decreased while the protein level of BCL-2 increased remarkably in the MI + AAV9 circSNRK group than MI + AAV9 ctrl group (Fig. 6B). Furthermore, circSNRK overexpression increased the proportion of proliferative cardiomyocytes (Fig. 6C). Next, we explored the effect of circSNRK overexpression on myocardial angiogenesis. Increased α-SMA^+^ and CD31^+^ arterioles in the border zone of the infarcted hearts at 14 days post-MI in rat was found in AAV9 circSNRK group compared AAV9 control group (Fig. 6D, E). These findings demonstrate that overexpression of circSNRK reduces cardiomyocyte apoptosis, promotes adult cardiac regeneration and angiogenesis in vivo.

### SNRK protects cardiomyocytes through GSK3β/β-catenin pathway

**Fig. 7:**
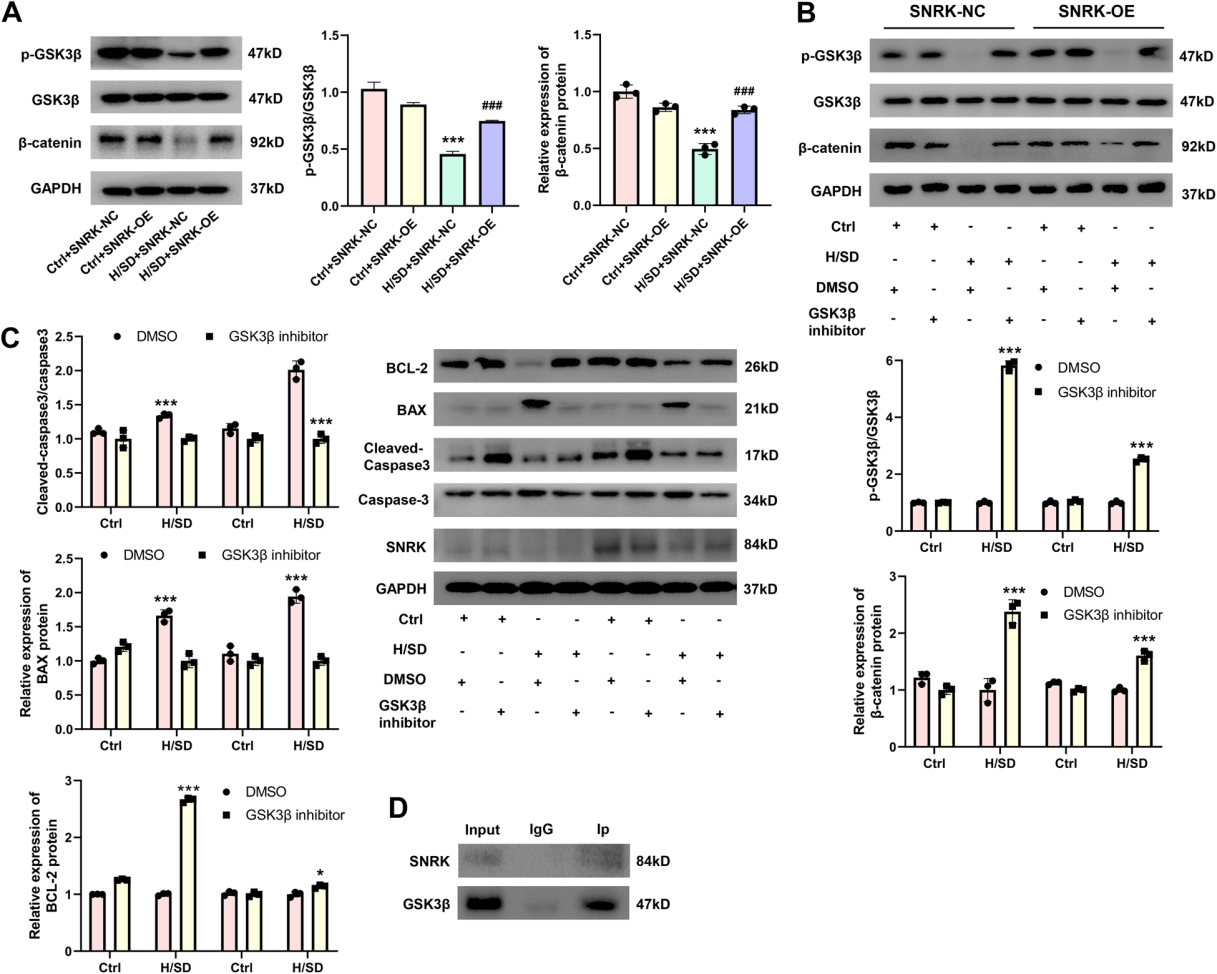
**SNRK protects cardiomyocytes through GSK3β/β-catenin pathway**. **A** Representative Western blot and quantified data show expression of GSK3β, phosphorylation of GSK3β, and β-catenin in hypoxic and ischemic NRCMs treated with SNRK overexpression ***p < 0.001 vs. ctrl + SNRK-NC group; ^###^*p* < 0.001 vs. H/SD + SNRK-NC group. (*n* = 3). **B** NRCMs were pretreated with DMSO or a GSK3β inhibitor for 2 h, followed by incubation with in hypoxic and ischemic condition for 12 h. Representative western blot and quantified data show expression of GSK3β, p-GSK3β, and β-catenin. ***p < 0.001 relative to DMSO in each group (*n* = 3). **C** Representative western blot and quantified data show expression of BCL-2, BAX, cleaved-caspase-3/caspase3. ***p < 0.001 relative to DMSO in each group (*n* = 3). **D** The interaction between SNRK and GSK3β in cardiomyocytes was assessed using immunoprecipitation and western blot analysis. Input, total cell lysates; IgG, immunoprecipitated with IgG.

We next sought to determine how SNRK overexpression alleviates hypoxia and ischemia induced heart injury. In vitro cardiomyocytes assays were used to determine whether it is involved in the apoptosis and proliferation. Previous study has revealed that GSK3β could regulate cardiomyocyte proliferation via degrading β-catenin^19^. Expression of p-GSK3β was significantly downregulated in H/SD conditions, but down-regulation of p-GSK3β was significantly lower in SNRK-OE transfected cardiomyocytes than in SNRK-NC transfected in H/SD conditions. Similarly, the decrease of β-catenin in H/SD conditions was markedly smaller in SNRK-OE transfected cardiomyocytes compared with the SNRK-NC transfected cardiomyocytes. Expression of β-catenin showed a significant increase in SNRK-OE transfected group compared with the SNRK-NC transfected group in H/SD conditions (Fig. 7A). Inhibition of GSK3β using GSK3β inhibitor activated the phosphorylation of GSK3β and then promoted the accumulation of β-catenin (Fig. 7B). At the same time, it abrogated the enhanced ratio of cleaved-caspase-3/caspase3 and the expression of BAX and the decreased expression of BCL-2 in cardiomyocytes (Fig. 7C), suggesting that SNRK modulates the phosphorylation of GSK3β that regulates apoptosis and proliferation. In addition, immunoprecipitation of SNRK followed by probing for GSK3β showing that SNRK is physically associated with GSK3β (Fig. 7D). All these results showed that the protective effects of SNRK may through GSK3β/β-catenin pathway.

## Discussion

Aberrant expression of circRNAs accounts for various cardiovascular diseases and circRNA can serve as a therapeutic target for diseases^20–22^. For example, knockdown of circRNA_101237 protected cardiomyocytes from apoptosis by sponging let‑7a‑5p, and that may be a potential therapeutic target for the management of cardiovascular diseases^23^. In the current study, results showed that circSNRK regulates cardiomyocyte apoptosis and proliferation by affecting the cardiomyocyte survival and cell cycle reentry processes both in vitro and in vivo, giving clear evidence that overexpression of circSNRK in cardiomyocytes conferred potent resistance to hypoxic and ischemic insults. Results further revealed that circSNRK overexpression could induce cardiomyocyte proliferation, which is important to promote functional recovery post MI^19,24^. Moreover, circSNRK significantly restored CD31+ and α-SMA+ artery density in the peri-infarcted zone of rats.

CircRNAs compete with linear mRNA and potentially act as sponges for miRNAs or proteins in order to regulate parental genes’ transcription or function as templates for translation^8,25^. Following biogenesis, circRNAs mainly locate in the cytoplasm with the exception of intron-containing circRNAs. Our results showed that circSNRK was expressed primarily in the cytoplasm of cardiomyocytes, suggesting a regulatory role at the post-transcriptional level. CircSNRK serves as a sponge for miR-103-3p, which had an inverse expression pattern in ischemic myocardium but directly bonded to circSNRK. Previous research has shown miR-103-3p is negatively linked to cardiomyocyte fate^26,27^. Importantly, circSNRK has greater efficiency in modulating miR-103-3p activity due to its abundance and stability. Finally, SNRK was verified as the target gene of miR-103-3p. Previous studies have revealed important roles of SNRK in cardiovascular development. Global homozygous SNRK knockout mice die at birth^28^, and SNRK deletion in the myocardium leads to heart failure and death by deteriorating mitochondrial efficiency and increasing mitochondrial uncoupling^29^. SNRK has been implicated as a repressor of inflammation^30^, regulates embryonic zebrafish vascular development^31^, and in mammals promotes angiogenesis in vivo^32^. Based on this evidence, we can conclude that SNRK participate in cell survival. Given that cardiac dysfunction is associated with cardiomyocyte survival post MI, it is not surprising that circSNRK/miR-103-3p/SNRK signaling is involved in cardiomyocyte survival. Furthermore, in the last rescue experiments, upregulating circSNRK or SNRK resulted in a reduction of apoptosis and promotion of proliferation of cardiomyocytes which were impaired by increasing levels of miR-103-3p. In this study, we revealed that the mRNA expression and protein level of SNRK were apparently increased by overexpressing circSNRK. These results all suggested that SNRK was modulated indirectly by circSNRK in cardiomyocytes.

The protective role of SNRK is likely associated with its interaction with GSK3β/β-catenin. Inhibition of GSK3β promotes cardiomyocyte proliferation in vitro^33,34^ and is cardioprotective in vivo^35^, and induces physiological hypertrophy^34^. Moreover, GSK3β inhibition in perfused rat hearts after ischemic preconditioning leads to the accumulation of β-catenin in the cytosol and nucleus^36^. GSK-3β stimulations are known to influence apoptosis and inhibit cell survival in the heart, whereas inhibition of GSK-3β downregulates apoptosis^37^. These results are consistent with previous studies in which the use of GSK3β inhibitor activated phosphorylation of GSK3β, promoted the accumulation of β-catenin, and abrogated the enhanced expression of apoptosis-associated proteins. SNRK levels inversely correlated with the H/SD-induced activity of apoptosis-associated gene. We also found overexpression of SNRK promoted phosphorylation of GSK3β, suggesting that SNRK inhibits H/SD-induced cardiomyocyte apoptosis and promotes proliferation by modulating the GSK3β/β-catenin pathway.

CircSNRK decreased fibrotic scar area nearly 55% and restored cardiac function, indicating an attractive therapeutic effect of attenuating cardiac remodeling post MI. Previous studies have indicated that SNRK has important roles in angiogenesis in vivo^32^. Results also revealed that circSNRK upregulation promotes angiogenesis after MI. Thus, the circSNRK/ miR-103-3p/ SNRK pathway might play a crucial role in regulating angiogenesis after MI.

This study has some limitations. First, although the effect of circSNRK on miR-103-3p function is evident and our results encouraging, we acknowledge that additional in vivo experiments are needed to further verify their function. Second, circSNRK was only overexpressed in one way, and more detailed loss-of-function experiments are required to further verify the role of circSNRK. Third, miR-103-3p has many target genes in addition to SNRK, while this study was focused on the function of SNRK. Finally, while the involvement of the GSK3β/β-catenin pathway was confirmed for cardiomyocyte apoptosis, whether it is involved in cardiomyocyte proliferation or angiogenesis resulting from hypoxia and ischemia reminds to be determined.

In summary, we demonstrated that overexpression of circSNRK inhibits cardiomyocyte apoptosis, induces cardiac regeneration and angiogenesis, and dramatically restored cardiac function and improved the prognosis post MI. This newly discovered circSNRK could be a promising therapeutic target for restoring cardiac function after MI.

## Materials and methods

### Circular RNA high-throughput sequencing

Total RNA was extracted from the border zone of sham or MI rat hearts (*n* = 4) and a RiboZero Magnetic Gold Kit was used to remove rRNA. A random priming method was utilized to amplify and transcribe the remaining RNAs into fluorescent cRNA (TruSeq SR Cluster Kit v3-cBot-HS, GD-401-3001, Illumina). The raw expression intensities were log2 transformed and normalized by Quantile normalization. The cutoffs were *p* ≤ 0.05 and |FC|≥ 1.5. Normality was assumed for log2 transformed normalized intensity values across samples per gene. Illumina HiSeq 4000 was used for high throughput circRNA sequencing to screen out differentially expressed circRNAs for verification by PCR.

### Animal model

All animal experiments were approved by the Animal Research Committee of the First Affiliated Hospital of Nanjing Medical University (No. IACUC-1905024) and followed the Guide for the Care and Use of Laboratory Animals published by the U.S. National Institutes of Health. And, in this study, we strictly adhered to all relevant ethical regulations. Eight-week-old wild-type male rats (Sprague-Dawley) were procured from experimental animal center of Nanjing Medical University. The animals were randomly assigned to different treatment groups and were blinded to the animal surgery and subsequent analysis for intervention. The details of constructing the rat MI model are as previously described^38^. Rats were intraperitoneally injected with 10% chloral hydrate aqueous solution (0.3 mL/100 g) for anesthesia, then fixed on a hardwood board in a supine position intubated and ventilated using the small animal ventilator. The rat’s left thorax was opened between the second and third ribs, and the left anterior descending coronary artery was quickly ligated 1 mm below the left atrial appendage. The color of the surrounding myocardial tissue changed from red to pale white, indicating a successful model. Finally, the chest was closed and penicillin was injected (100,000 units) intramuscularly to prevent infection. In the sham group, only threading was performed without ligation.

### Isolation and culture of neonatal rat cardiomyocytes

Primary cardiomyocytes were isolated from 1-day-old (P1) S/D rats. Heart ventricles of neonatal pups were minced into one mm^3^ pieces, and then digested with a solution containing 0.25% trypsin and 0.1% collagenase II. The dissociated cells were pre-plated at 37 °C for 1.5–2 h. After that, using differential adhesion, cardiomyocytes were separated from fibroblasts. The cardiomyocytes were cultured with Dulbecco’s modified Eagle’s medium (DMEM; Gibco, New York, USA) containing 5% fetal bovine serum (FBS, Gibco, New York, USA) and 10% horse serum (HS, Gibco, New York, USA) at 37 °C in a 5% CO_2_ incubator.

### Transfection

After 1 day of pre-culture, the primary cardiomyocytes were transfected with plasmids according to the specifications for Lipofectamine 2000 (Invitrogen, California, USA). At 6 h after transfection, the cells were cultured in fresh medium. MiRNA mimics (100 nmol/L), miRNA inhibitor (100 nmol/L), and their negative controls (100 nmol/L) were carried out using riboFECTTM CP Reagent (Ribobio, Guangzhou, China) to incubate 20 h according to the manufacturer’s instructions. Then, cells were placed in either hypoxic and serum deprivation (H/SD) or normoxic conditions for another 36 h.

### RNA extraction and RNase R treatment

Total RNA was extracted from isolated primary cardiomyocytes using Trizol reagent (Life, New York, USA). The extracted RNA was quantified by a NanoDrop 2000 (Thermo Fisher, New York, USA), and incubated with RNase R (3 units of RNase for 1 mg RNA; Epicentre, Wisconsin, USA) for 15 min at 37 °C. Phenol-chloroform was then used to purify the extracted RNA and then re-precipitated in ethanol.

### QRT-PCR

For quantification of circSNRK, miR-103-3p, or SNRK mRNA, PrimeScript™RT reagent Kit with gDNA Eraser (Vazyme biotech, Nanjing, China) was used to synthesize the first-strand cDNA. Stem-loop qRT-PCR was performed using a FastStart Essential DNA Green Master (Vazyme biotech, Nanjing, China). GAPDH was used to normalize the expression of circSNRK and SNRK mRNA. For quantification of miR-103-3p, Bulge-loop^TM^ miRNA qRT-PCR Primer Sets (one RT primer and a pair of qPCR primers for each set) specific for miR-103-3p is designed by RiboBio (Guangzhou, China), cDNA was synthesized with a miRNA 1st Strand cDNA Synthesis Kit (by stem-loop) (Vazyme biotech, China). AceQ qPCR SYBR Green Master Mix (Vazyme biotech, Nanjing, China) was then used for real-time PCR. U6 was used to normalize the cellular miR-103-3p expression. The formula was: relative gene expression = 2^−ΔΔCt^. The primers are listed in Supplementary Table 1.

### Western blot analysis

Lysis buffer was used to extract total proteins from the LV infarct border zone or cells. Subsequently, the BCA protein detection kit was used to determine the total protein concentration (KeyGEN BioTECH, Shanghai, China). The total protein (20 μg) was electrophoresed and analyzed using primary antibodies such as BAX (5023), caspase3 (9662), cleaved-caspase-3 (9664), β-catenin (8480), Gsk3β (12456), p-Gsk3β (5558), and GAPDH (5174) all purchased from Cell Signaling Technology, USA. BCL-2 (ab196495) was purchased from Abcam, Cambridge, England). The next day, horseradish peroxidase-conjugated secondary antibodies (Santa Cruz, California, USA) were used to bind primary antibodies. The bands were visualized using enhanced chemiluminescence agents and protein expression analysis was performed using a gel documentation system (Bio-Rad Gel Doc1000 and Multi-Analyst version 1.1).

### Luciferase assay

HEK-293T cells were cultured at 5% CO_2_, 37 °C. HEK-293T cells were co-transfected with miR-103-3p mimics and miR-103-3p-NC (50 nM; Ribobio, Guangzhou, China), and a reporter plasmid containing the 3′ UTR of circSNRK (250 ng/well) (GeneChem, Shanghai, China) inserted downstream of the luciferase reporter gene (PGL3-circSNRK-UTR) were transfected by Lipofectamine 2000 in a 96-well plate. 24 h after transfection, SV-Renilla luciferase plasmids (5 ng/well) was used as the internal control. Cells were collected after 24 h transfection, and luciferase activity was detected according to the instructions of the Dual Luciferase Reporter Assay kit (Promega, Wisconsin, USA).

### Apoptosis assay

Control plasmids or circSNRK-overexpressed plasmids were used to treat primary cardiomyocytes for 8 h. After washing with PBS, cells were stained with Annexin V-fluoresce in isothiocyanate (V-FITC) and propidium iodide (PI) apoptosis kit (KeyGen Biotech, Shanghai, China). The cells were then treated with hypoxia and serum starvation for 12 h. Next, TdT-mediated dUTP nick end labeling (TUNEL) staining was used to assess apoptosis rate. In addition, the Cell Counting Kit-8 (CCK-8; Dojindo, Japan) was used to detect the apoptosis following the manufacturer’s instructions. Primary cardiomyocytes (5,000 cells/well) were seeded in 96-well plates and 10 μL CCK-8 solution was added to each well. After 2 h, synergy 2 microplate reader (Bioteck, Vermont, USA) was used to detect the absorbance at 450 nm.

### AAV9 mediated circSNRK delivery in vivo

Rats were randomly divided into 4 groups including sham + AAV-NC group (*n* = 6), sham + AAV-circSNRK group (*n* = 6), MI + AAV-NC group (*n* = 6), and MI + AAV-circSNRK group (*n* = 6). In order to optimize the cardiac circSNRK overexpression, we injected AAV9 mediated gene delivery into the myocardium. 20 μL containing 5 × 10^10^ GC (genome copies) of AAV9 vectors were injected in left ventricles one week before the establishment of the rat MI model.

### Echocardiography

Rats were anesthetized with 100 mg/kg ketamine combined with 10 mg/kg xylazine inhalation with an isoflurane delivery system (Viking Medical, Medford, USA). Transthoracic two-dimensional M-mode echocardiogram was obtained using 2000 high resolution micro imaging system (Visual Sonic) equipped with 30 MHz transducer. Echocardiographic studies were performed at 1 day, 2 and 4 weeks post MI on rats. The internal diameter of the left ventricular was measured in the parasternal long-axis view from M mode recordings. Left ventricular end-diastolic dimension (LVEDD) and left ventricular end systolic dimension (LVESD) were measured in at least three consecutive cardiac cycles. Left ventricular ejection fraction (LVEF) and left ventricular fractional shortening (LVFS) were used to evaluate the systolic function of the hearts. Corresponding formulas as: EF (%) = [(EDD^3^ − ESD^3^) /EDD^3^] × 100; FS (%) = [EDD − ESD)/EDD] × 100. All measurements were made by an independent blinded sonographer.

### Morphometric studies

The heart was fixed with 4% paraformaldehyde. At room temperature, the prepared heart sections were first stained with Weigert’s iron hematoxylin solution for 5 min and Biebrich Scarlet-acid Fuchsin solution for 2 min. Aniline Blue reverse staining for 5 min and 1% acetic acid for 2 min. Finally, image-J software was used for morphological analysis of Masson’s trichrome staining tissue sections, including infarct area and fibrosis percentage (NIH, version 1.30). The heart was cut into 5 pieces using a surgical carbon steel razor blade. Place the heart sections in 1% 2,3,5-triphenyltetrazolium chloride (TTC) for 15 min at room temperature. 4% paraformaldehyde was used to fix stained heart slices overnight at 4 °C. Photograph heart sections and measure infarct area on image-J software.

### Immunocytochemical staining

Rat heart slides were incubated in blocking buffer for 2 h. Then, the primary antibodies diluted in blocking buffer were used to incubate cell at 4 °C overnight. PBS was used to wash the cells three times, and the corresponding secondary antibody was diluted in the blocking buffer solution and used for staining at room temperature for 1 h. Stained slides were then rinsed and mounted in a mounting medium containing DAPI (YeaSen, China). Fluorescence microscope (Carl Zeiss, Thuringia, Germany) was used to observe the images. Primary antibodies and dilutions were used in immunocytochemical staining (ICC) including anti-Ki67 antibody (Abcam) and anti-α-actinin (Abcam). Secondary antibodies and dilutions used in ICC included goat anti-rabbit IgG2a Alexa Fluor 488 (Yeasen 33106ES60, 1:100, Shanghai, China) and goat anti-mouse Cy3 (Yeasen 33208ES60, 1:100, Shanghai, China).

### Histology

To analyze cell apoptosis in vivo, the TdT UTP nick end-labeling (TUNEL) in situ cell death detection kit (11684795910, Roche, Switzerland) was used to stain the myocardial tissue sections. The proliferation of cardiomyocytes was assessed by Ki67 (12075, Cell Signaling Technology, USA, 1:100). A new capillary network was detected by CD31 (AF3628, R&D systems, 1:100) and α-SMA (A2547, Sigma Aldrich, 1:250) staining. Nuclei were counter-stained with DAPI (Sigma Aldrich, St Louis, 1:10,000) and sections were observed with a fluorescent inverted microscope (Carl Zeiss, Thuringia, Germany).

### Statistics

Data are expressed as mean ± SD and percentages (%), respectively. For continuous variables, Student *t* test (normal distribution data) was used between two groups. One-way ANOVA (three or more than three groups) followed by Bonferroni’s correction, if needed, were performed. All statistical tests were performed with Prism 8.1.2 (GraphPad Software Inc.), and *P* < 0.05 (two-sided) was considered statistically significant.

## Supplementary information

supplementary table

supplementary table legends

supplementay figure legends

supplementary Fig-S1

supplementary Fig-S2

supplementary Fig-S3

## Data Availability

Data supporting present findings are available from the corresponding author upon reasonable request.
