# Supplementary material for: Overexpression of circRNA SNRK targets miR-103-3p to reduce apoptosis and promote cardiac repair through GSK3β/β-catenin pathway in rats with myocardial infarction
Source: Cell Death Discov. 2021 Apr 19;7:84. doi: 10.1038/s41420-021-00467-3 (PMC8055694; doi:10.1038/s41420-021-00467-3)
Supplement: Supplementary file 1 — supplementary table [file 41420_2021_467_MOESM1_ESM.docx]

| Gene name | Forward (5'>3') | Reverse (5'>3') |
| --- | --- | --- |
| circSNRK(divergent)  circSNRK(convergent)  circMyocd  circSmad1  circNf2  circGigyf2  SNRK  U6  GAPDH (divergent)  GAPDH (convergent) | CAGCCTGGAAAGAAGCTCACT  TGCTCAGATAGTTCACGCTATATCC  TCCGTGAAAGAGGCTATAAAAGTCT  ACTATAAGCGAGTGGAGAGCCC  TTGGATGAAAAGGTCTATTGCC  AAACCAGGAAGAAAAGATGTAGGCT  TCGTAGAAGCCCTGGAAA  AGAGAAGATTAGCATGGCCCCTG  GAATACCATCAATAAAGTTCGCTG  ACAACTTTGGTATCGTGGAAGG | GCAAAACTGCATGACAGATCACT  CATTCTCTGGTTTCAAGTCTCTGTG  TCCAGTTTTTTCCTCGGGTC  GTTCGGAAAGGTAGGAAATACTGTT  AAAGAACCAGGTCTCCCGAA  CTAACATTTCTTCTCGGCCATATC  AACTGGGCCTTGATGTTG  AGTGCAGGGTCCGAGGTATT  GTCCGATACGGCCAAATCC  GCCATCACGCCACAGTTTC |
| RT-PCR, quantitative reverse-transcription polymerase chain reaction | | |

**Supplementary table 1.**
