# Supplementary material for: Overexpression of circRNA SNRK targets miR-103-3p to reduce apoptosis and promote cardiac repair through GSK3β/β-catenin pathway in rats with myocardial infarction
Source: Cell Death Discov. 2021 Apr 19;7:84. doi: 10.1038/s41420-021-00467-3 (PMC8055694; doi:10.1038/s41420-021-00467-3)
Supplement: Supplementary file 2 — supplementary table legends [file 41420_2021_467_MOESM2_ESM.docx]

**Supplementary table legends**

**Supplementary Table 1. Sequences of primer.**
