# Supplementary material for: Overexpression of circRNA SNRK targets miR-103-3p to reduce apoptosis and promote cardiac repair through GSK3β/β-catenin pathway in rats with myocardial infarction
Source: Cell Death Discov. 2021 Apr 19;7:84. doi: 10.1038/s41420-021-00467-3 (PMC8055694; doi:10.1038/s41420-021-00467-3)
Supplement: Supplementary file 3 — supplementay figure legends [file 41420_2021_467_MOESM3_ESM.docx]

**Supplementary Figure legends**

**Fig. S1.** (A) The circSNRK nucleotide sequence is strongly conserved, with >85% homology among humans and rats. (B) qRT-PCR analysis of miR-103-3p in heart tissue of Sham and MI groups. ***p<0.001 vs. Sham (n = 6).

**Fig. S2 miR-103-3p elevation aggravates H/SD induced cardiomyocyte apoptosis and reduced cardiomyocyte proliferation.** (A) qRT-PCR analysis of miR-103-3p in miR-NC and miR-103-3p treated primary cardiomyocytes. ***p<0.001 *vs.* miR-NC (n = 6). (B-D) Cardiomyocytes were transfected with miR-NC and miR-103-3p mimics in H/SD conditions. MiR-103-3p mimics promoted apoptosis by flow cytometry analysis (n = 3) (B) and TUNEL analysis. (n = 3) White arrows indicate apoptotic cells. bars = 50 μm. (C) and decreased cell viability by Cell Counting Kit-8 (CCK-8) assay (n = 6) (D) ***p<0.001 *vs.* Ctrl+ miR-NC, ^###^p<0.001 *vs.* H/SD + miR-NC (n = 6) (E) BCL-2, BAX, cleaved-caspase-3/caspase-3 levels after overexpression of miR-103-3p in cardiomyocytes. *P<0.05, ***p<0.001 *vs.* Ctrl + miR-NC, ^##^P<0.01, ^###^p<0.001 *vs.* H/SD + miR-NC (n = 3) (F) EdU staining in isolated primary cardiomyocytes transfected with miR-NC and miR-103-3p mimics and quantification of EdU positive primary cardiomyocytes. White arrows indicate EdU positive CMs. ***p<0.001 **P<0.01 *vs.* miR-NC; bar = 50 µm. (n = 6). (G) Ki67 immunofluorescence staining in isolated primary cardiomyocytes transfected with miR-NC and miR-103-3p mimics and quantification of Ki67 positive primary cardiomyocytes. White arrows indicate Ki67-positive CMs. ***p<0.001 *vs.* miR-NC; bar = 50 µm. (n = 6). (H) Flow cytometry analysis of primary cardiomyocytes transfected with miR-NC and miR-103-3p mimics. *P<0.05, **p<0.01 *vs.* miR-NC group (n = 3).

**Fig. S3 Loss of miR-103-3p ameliorates hypoxia induced cardiomyocyte apoptosis and increased cardiomyocyte proliferation.** (A-C) Primary cardiomyocytes were transfected with miR-NC and miR-103-3p inhibitor in H/SD conditions. MiR-103-3p inhibitor decreased apoptosis by flow cytometry analysis (n = 3) (A) and TUNEL analysis White arrows indicate apoptotic cells. bar = 50 μm (B) and increased cell viability by Cell Counting Kit-8 (CCK-8) assay (n = 6) (C) ***p<0.001 *vs.* Ctrl + miR-NC, ^#^P<0.05, ^###^p<0.001 *vs.* H/SD + miR-NC (n = 6) (D) BCL-2, BAX, cleaved-caspase-3/aspase-3 levels after downregulation of miR-103-3p in hypoxic and ischemic primary cardiomyocytes. ***p<0.001 *vs.* Ctrl + miR-NC, ^#^P<0.05 *vs.* H/SD + miR-NC (n = 3) (E) EdU staining in isolated primary cardiomyocytes transfected with miR-NC and miR-103-3p inhibitor and quantification of EdU positive CMs. White arrows indicate EdU positive CMs. ***p<0.001 *vs.* miR-NC; bar = 50 µm. (n = 6). (F) Ki67 immunofluorescence staining in isolated primary cardiomyocytes transfected with miR-NC and miR-103-3p inhibitor and quantification of Ki67 positive primary cardiomyocytes. White arrows indicate Ki67-positive primary cardiomyocytes. ***p<0.001 *vs.* miR-NC; bar = 50 µm. (n = 6). (G) Flow cytometry analysis of primary cardiomyocytes transfected with miR-NC and miR-103-3p inhibitor. *P<0.05 ***p<0.001 *vs.* miR-NC group (n = 3).
