# Supplementary figures and images for: Overexpression of circRNA SNRK targets miR-103-3p to reduce apoptosis and promote cardiac repair through GSK3β/β-catenin pathway in rats with myocardial infarction
Source: Cell Death Discov. 2021 Apr 19;7:84. doi: 10.1038/s41420-021-00467-3 (PMC8055694; doi:10.1038/s41420-021-00467-3)

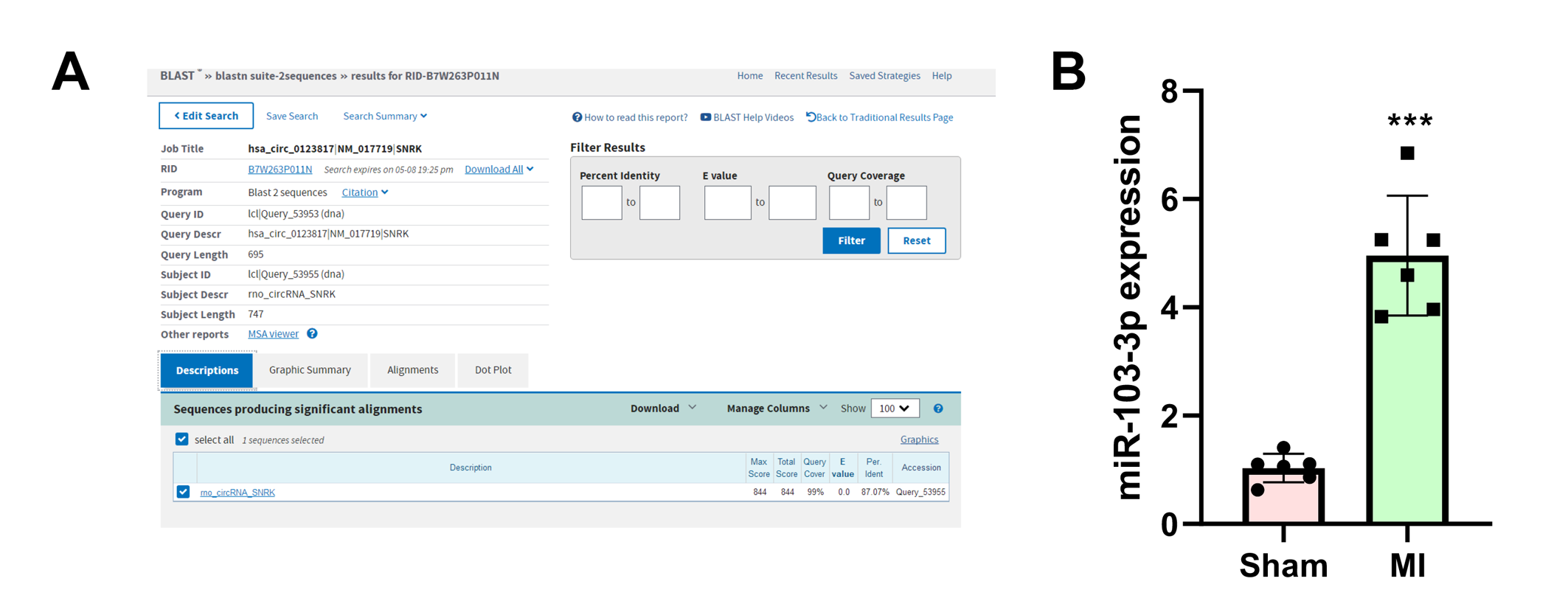

Supplement: Supplementary file 4 — supplementary Fig-S1 [file 41420_2021_467_MOESM4_ESM.tif]

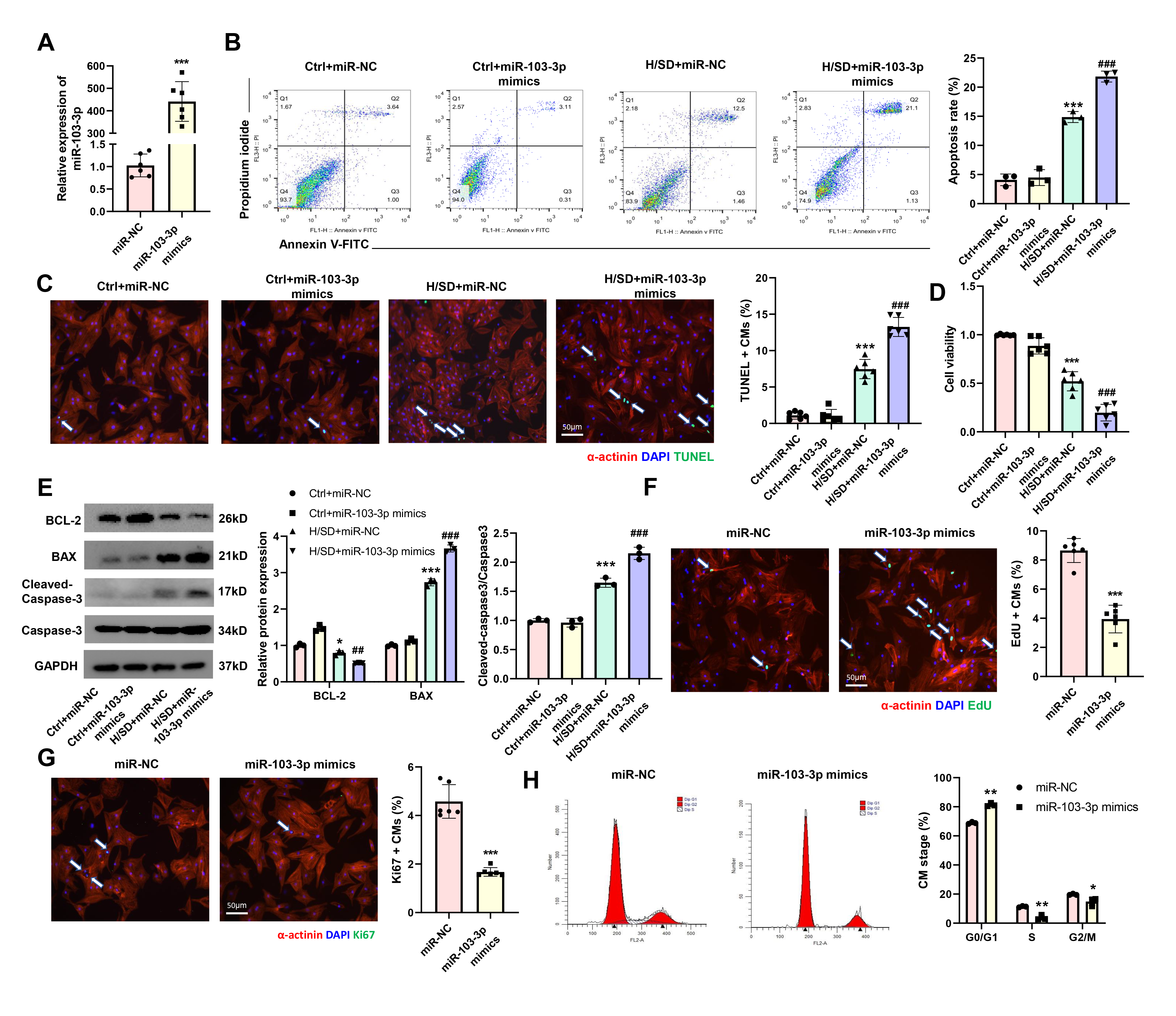

Supplement: Supplementary file 5 — supplementary Fig-S2 [file 41420_2021_467_MOESM5_ESM.tif]

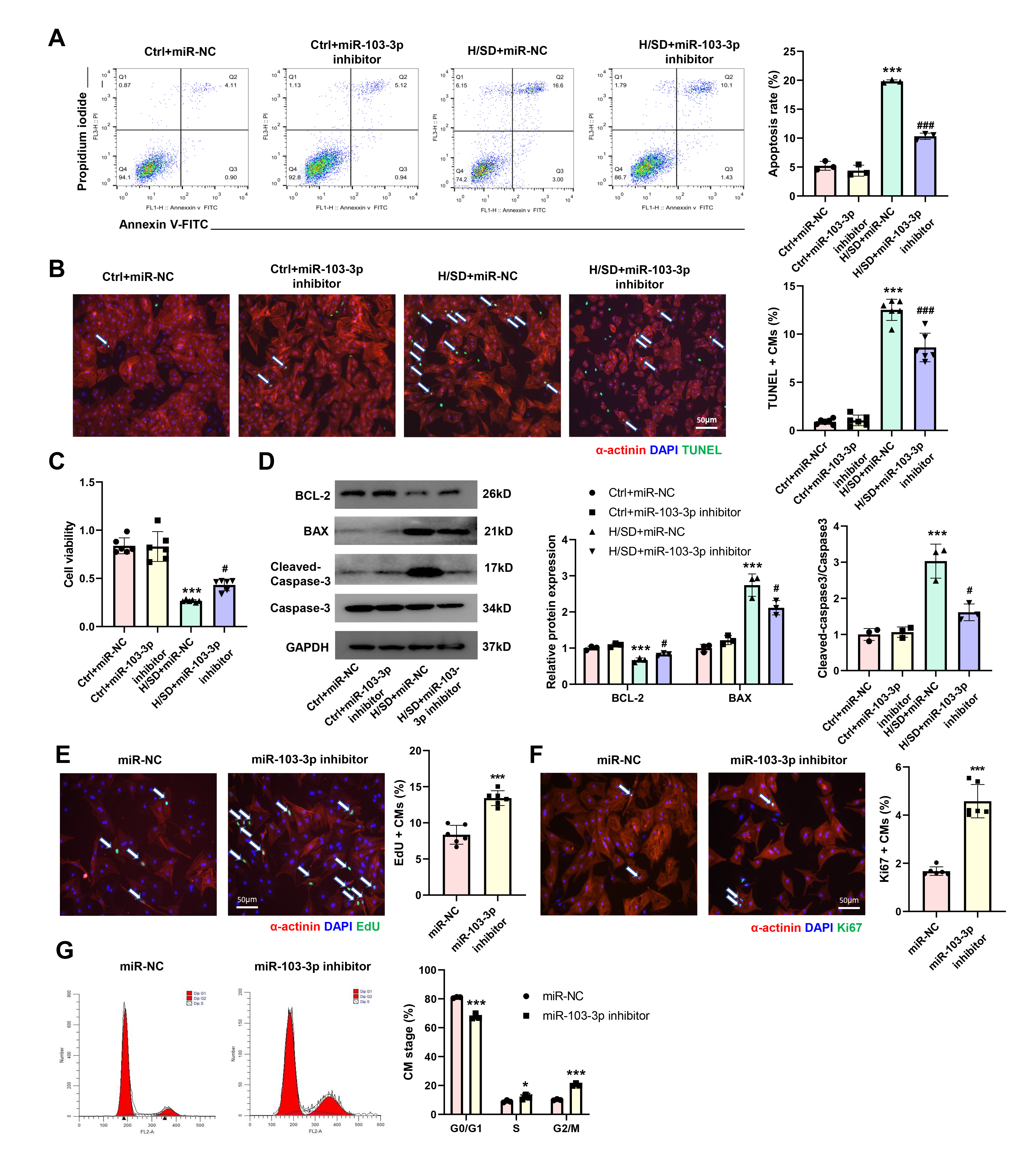

Supplement: Supplementary file 6 — supplementary Fig-S3 [file 41420_2021_467_MOESM6_ESM.tif]
